# Parotid Gland Magnetic Resonance Elastography Feasibility Study: Clinical Diagnostic Potential and Future Perspectives as a Radiological Palpation Method

**DOI:** 10.3390/diagnostics15182351

**Published:** 2025-09-16

**Authors:** Merve Solak, Esat Kaba, Mehmet Beyazal, Metin Çeliker, Fatma Beyazal Çeliker

**Affiliations:** 1 Department of Radiology, Recep Tayyip Erdogan University, Rize 53100, Türkiye; esatkaba04@gmail.com (E.K.);; 2 Department of Otorhinolaryngology, Recep Tayyip Erdogan University, Rize 53100, Türkiye

**Keywords:** parotid gland, tissue stiffness, head and neck, magnetic resonance elastography, radiological palpation

## Abstract

**Background/Objectives:** Magnetic resonance elastography (MRE) is a noninvasive imaging technique that quantitatively characterizes tissue mechanical properties. This study aimed to establish and validate a feasible parotid MRE protocol using 3T MRI, with potential relevance for oral and maxillofacial surgery (OMFS) and otorhinolaryngology practice. **Methods:** This study included 21 healthy volunteers (18 women, 3 men; mean age, 49 years) examined between January and May 2024. MRE was performed using a 3.0 Tesla MRI system (Discovery MR750w, GE Healthcare, Waukesha, WI, USA) with a passive driver positioned over the parotid gland in a 16-channel head/neck coil. Two radiologists independently analyzed axial magnitude images drawing regions of interest (ROIs) encompassing the entire gland while excluding intraparotid lymph nodes and vascular structures. Mean and maximum stiffness values (kPa) were recorded for each gland. Interobserver agreement was assessed using intraclass correlation coefficients (ICCs) and Bland–Altman analysis. **Results:** Mean stiffness was 1.209 ± 0.240 kPa (Radiologist 1) and 1.146 ± 0.233 kPa (Radiologist 2); maximum stiffness was 1.595 ± 0.532 kPa and 1.563 ± 0.528 kPa, respectively. ICCs were 0.638 for mean stiffness and 0.918 for maximum stiffness, indicating moderate-to-excellent agreement. **Conclusions**: MRE is a technically feasible and reproducible method for evaluating parotid stiffness using standard imaging infrastructure. This feasibility study in healthy volunteers provides normative stiffness values for the parotid gland and supports MRE as a potential tool for “radiological palpation” to aid in the differentiation of salivary gland lesions and post-treatment assessment in OMFS and otorhinolaryngology practice.

## 1. Introduction

The parotid glands are the largest salivary glands in the human body [1,2]. They are the most common site for benign salivary gland tumors and the second most common site for malignant salivary gland tumors [3,4]. In the diagnosis of parotid gland pathologies, otolaryngology specialists rely on both physical examination and imaging findings [5]. Ultrasonography (US) is the primary imaging modality for the parotid glands; however, magnetic resonance imaging (MRI) has become increasingly important due to its superior soft tissue contrast and spatial resolution [4,5]. Various qualitative and quantitative MRI techniques have been applied to characterize parotid gland pathologies, among which magnetic resonance elastography (MRE) represents a notable innovation [6,7].

Elastography is a non-invasive imaging technique that objectively evaluates the biomechanical properties of tissues. First introduced in the late 1980s using ultrasound, it has since been widely applied in clinical practice across multiple organs [1,5]. MRE visualizes mechanical wave propagation within tissues and quantitatively measures tissue stiffness, offering an innovative diagnostic perspective [8,9]. Effective implementation of MRE requires shear wave generation, displacement detection, and conversion of these data into stiffness maps [10,11,12]. Despite its success in the liver, breast, and brain, studies investigating MRE in the head and neck region—particularly the parotid gland—are limited [11,13,14]. This gap highlights the need for standardized parotid specific MRE protocols and normative stiffness data, which could provide valuable quantitative parameters for oral and maxillofacial surgery (OMFS) and otorhinolaryngology practice.

The primary objective of this study was to develop an optimized MRE protocol for the head and neck region and to assess its feasibility for evaluating the parotid gland using a 3.0 Tesla MRI system. This approach aimed to establish parotid gland–specific stiffness values in a healthy population. The data obtained from this study may serve as a reference for future research focused on characterizing parotid gland pathologies, guiding treatment planning in OMFS, and assessing potential post-radiotherapy complications in the head and neck region. Furthermore, the developed protocol has the potential to provide a novel diagnostic and prognostic perspective for integrating parotid gland MRE into clinical practice.

## 2. Materials and Methods

**Ethics:** This single-center study was conducted in the radiology clinic. Consent was obtained from all participants in this study. It was approved by the Ethical Review Committee of Recep Tayyip Erdoğan University Training and Research Hospital (Decision Number: 2024/295, approved date: 12 December 2024).

### 2.1. Study Design and Participants

This feasibility study was conducted in January 2024 using a 3.0 Tesla MRI scanner (GE Discovery MR750w, GE Healthcare, Waukesha, WI, USA) equipped with a dedicated 16-channel head/neck coil. Initially, 24 participants were recruited; three were excluded due to inadequate penetration of shear waves caused by motion-related or susceptibility artifacts, ensuring the reliability of subsequent quantitative analysis. The final study population comprised 21 participants (18 females, 3 males; mean age, 49 years; range, 20–83 years).

### 2.2. Imaging Protocol and MRE Technique

Participants were voluntarily enrolled from routine outpatient cases who underwent brain MRI for various clinical indications. Inclusion criteria included: no prior history of radiotherapy to the head and neck region, no history of intracranial surgery, and no history of parotid gland surgery or known parotid pathology. All participants provided written informed consent, and elastography was performed immediately following routine brain MRI without altering the standard imaging workflow.

All examinations were performed with participants in the supine position. For anatomical evaluation, the standard brain MRI protocol was first acquired, including three-plane localizer images, followed by high-resolution axial fat-suppressed T1-weighted images (repetition time [TR], 6.91 ms; echo time [TE], 2.63 ms; field of view [FOV], 240 mm; slice thickness, 1.2 mm; number of slices, 134) to delineate the head and neck anatomy in detail.

MRE was subsequently performed according to the protocol summarized in Table 1. A commercially available pneumatic liver MRE driver was adapted for parotid gland imaging. A 2D MRE protocol was chosen for its clinical availability, shorter acquisition time, and proven robustness, providing a practical. Only a single parotid gland was imaged per participant to optimize signal quality and patient comfort, recognizing that this precludes direct bilateral comparison. The side for MRE acquisition was chosen according to patient comfort; if no preference was indicated, the side was randomly assigned, ensuring unbiased selection independent of clinical findings. The driver was positioned externally over either the left or right parotid gland, within the head coil, using gentle but firm direct compression. A piston extension transmitted 60 Hz mechanical vibrations through the overlying soft tissues into the parotid parenchyma. Proper coupling and the absence of air gaps between the driver surface and the skin were visually confirmed prior to acquisition.

### 2.3. Image Postprocessing and ROI Analysis

The MRE datasets were retrospectively evaluated by two independent readers: a radiology resident and a board-certified radiologist with experience in head and neck imaging. Regions of interest (ROIs) were manually delineated on axial magnitude images to precisely encompass the parotid gland tissue, and these ROIs were subsequently transferred onto the corresponding stiffness maps. To ensure accurate quantification, the ROIs were further verified and adjusted based on confidence maps, which help delineate regions with reliable elastographic data. Polygonal two-dimensional ROIs were carefully placed on a single axial slice displaying the largest cross-sectional area of the visible parotid gland, with deliberate exclusion of intraparotid lymph nodes, adjacent vascular structures, and any image artifacts that could compromise measurement accuracy.

For each participant, both the mean stiffness value of the entire delineated gland and the maximum stiffness value—derived from the color-coded elastograms representing peak tissue rigidity—were recorded. The average ROI size was 2.1 ± 0.6 cm^2^, and distinct ROIs were employed for the determination of mean and maximum stiffness values in line with established imaging protocols. Histogram-based or voxel-wise distribution analyses were not conducted due to inherent limitations in spatial resolution and slice thickness in the MRE acquisitions of this anatomically complex region.

The ROI placement and stiffness quantification process were iteratively refined using confidence maps, which facilitated the exclusion of pixels affected by noise or low signal-to-noise ratio, thereby enhancing the reliability of the stiffness measurements within the glandular tissue.

### 2.4. Statistical Analysis

Interobserver reliability between the two readers was quantitatively assessed using the intraclass correlation coefficient (ICC), computed separately for mean and maximum stiffness measurements. The degree of agreement was further illustrated using Bland–Altman plots, which graphically represent the limits of agreement and systematic biases, if any, between the observers.

Due to the study’s design involving a single time-point acquisition, test–retest repeatability assessments were not performed. Differences between the two observers’ measurements were statistically analyzed using two-sample independent t-tests to detect any significant measurement bias. A significance threshold of *p* < 0.05 was adopted for all statistical tests. All analyses were executed using SPSS version 26 software (IBM Corp., Armonk, NY, USA).

**Figure 1:**
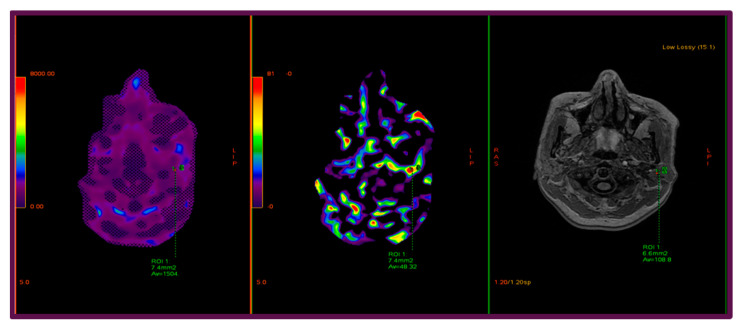
Representative images obtained using a 3.0 Tesla MRE system from a 45-year-old female participant illustrate stiffness measurement of the left parotid gland. The images are presented from left to right as follows: confidence map, stiffness map (elastogram), and axial fat-unsuppressed T1-weighted anatomical reference image. Regions of interest (ROIs) were manually delineated as polygonal contours encompassing the entire parotid gland while deliberately excluding vascular structures and intraglandular lymph nodes. Quantitative stiffness values, expressed in kilopascals, were derived from the central slice of the elastogram. Measurement reliability was ensured through the evaluation of the confidence map, while precise ROI placement was guided by the corresponding anatomical T1-weighted images.

**Figure 2:**
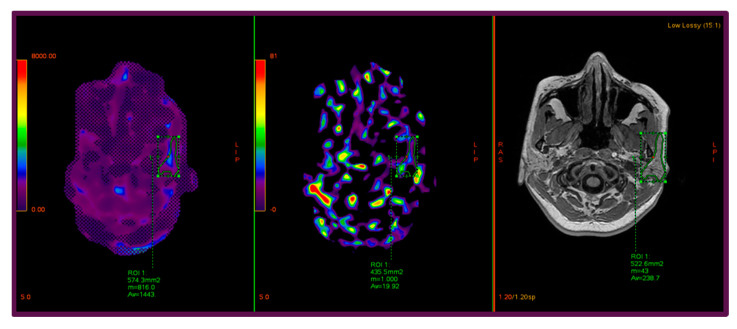
Axial 3.0 Tesla MRE images of the parotid gland from a 53-year-old female participant, illustrating a focused quantitative assessment of tissue stiffness. From left to right, the confidence map, stiffness map (elastogram), and corresponding axial T1-weighted anatomical image are presented. The entire gland was systematically examined on the elastogram to identify the region with the highest stiffness signal intensity. A minimally sized, manually delineated region of interest (ROI) was precisely placed over the area exhibiting maximal and homogeneous stiffness, with rigorous cross-referencing against both anatomical and confidence maps to ensure accuracy. Care was taken to exclude partial volume artifacts and confounding structures, ensuring the ROI encompassed only parenchymal tissue.

To illustrate the methodology and highlight the quantitative assessment of parotid stiffness, Figure 1 presents representative images from a 45-year-old female participant, showing the confidence map, stiffness map, and corresponding anatomical T1-weighted image, with manually delineated ROIs encompassing the gland while excluding vascular and lymphatic structures. Similarly, Figure 2 illustrates the parotid gland of a 53-year-old female participant, highlighting the region of maximal homogeneous stiffness. The confidence map, stiffness map, and axial T1-weighted image are shown side by side, and a carefully delineated ROI was placed to include only parenchymal tissue, avoiding vascular structures and partial volume artifacts, with cross-reference to both confidence and anatomical maps to ensure accuracy.

## 3. Results

A total of 21 healthy participants were included in this study. MRE was performed on the left parotid gland in 11 participants and on the right parotid gland in 10 participants. The mean age of subjects in the left parotid group was 55.36 ± 15.29 years, whereas the right parotid group had a mean age of 43.60 ± 14.58 years.

Quantitative shear stiffness measurements of the parotid glands were successfully acquired in all participants without technical failure. Both maximum and mean stiffness values were independently assessed by two radiologists (designated as Radiologist 1 [R1] and Radiologist 2 [R2]). All values are expressed in kilopascals (kPa) and are summarized in the corresponding tables.

Table 2 presents the overall stiffness measurements across the cohort. Mean stiffness tended to be slightly higher in the left parotid glands (R1_mean: 1.356 ± 0.190 kPa) compared to the right side (R1_mean: 1.118 ± 0.167 kPa). Independent two-sample *t*-tests confirmed that this difference was statistically significant (*p* = 0.041). Maximum stiffness values showed a similar trend, although the small cohort size limits definitive conclusions regarding side-specific differences.

Table 3 and Table 4 provide stratified analyses according to sex. Table 3 presents the stiffness measurements of the single male participant; these values are reported individually without measures of variance due to the lack of replication, rendering standard deviations inapplicable (N/A). Table 4 summarizes the stiffness measurements for female participants, which demonstrated marginally lower mean and maximum stiffness values compared to the overall cohort.

Descriptive statistics for the entire study population are detailed in Table 5. The inter-rater agreement, evaluated using the intraclass correlation coefficient (ICC), was 0.638 for mean stiffness and 0.918 for maximum stiffness values. The ICC for mean stiffness indicates moderate agreement, whereas the ICC for maximum stiffness reflects excellent concordance between the observers, supporting the acceptable reproducibility of MRE-derived stiffness measurements in this application.

To place our findings in context, Table 6 summarizes published studies on parotid elastography, highlighting differences in methodology, imaging hardware, and reported stiffness values. This comparison supports the technical feasibility of parotid MRE and emphasizes the need for standardized protocols.

**Figure 3:**
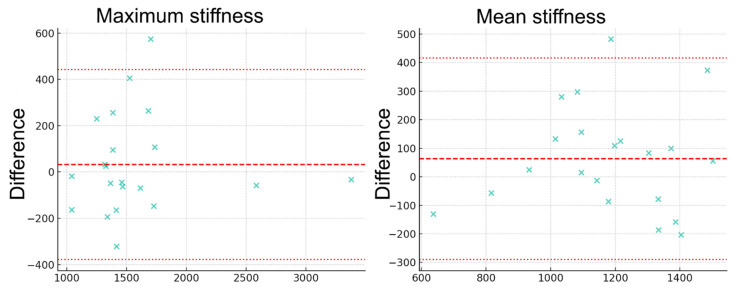
Bland–Altman plots illustrating interobserver agreement for mean and maximum stiffness measurements obtained from magnetic resonance elastography. The majority of data points (green crosses) lie within the 95% limits of agreement (dotted red lines), with only a single participant exhibiting values outside these boundaries in both measurement categories. This demonstrates a high degree of concordance and reproducibility between the two independent radiologists.

Bland–Altman plots (Figure 3) further illustrate the interobserver agreement, with only one participant’s measurements falling outside the limits of agreement for both mean and maximum stiffness values, underscoring the stability and reliability of the quantification.

Additionally, independent two-sample t-tests revealed no statistically significant differences between the stiffness measurements obtained by the two radiologists (*p* = 0.85 for mean stiffness and *p* = 0.39 for maximum stiffness), reinforcing the robustness of the inter-rater reliability.

## 4. Discussion

Magnetic resonance imaging remains the gold standard modality for the evaluation of suspected neoplastic lesions within the parotid gland, due to its superior soft tissue contrast resolution and detailed anatomical visualization capabilities [13,14,17]. MRI enables precise assessment of tumor location, extent of local invasion, perineural spread, and spatial relationships with adjacent anatomical structures, which are crucial for accurate diagnosis and treatment planning [18,19]. Recent advancements in MRI techniques, including diffusion-weighted imaging (DWI), dynamic contrast-enhanced MRI (DCE-MRI), and magnetic resonance spectroscopy, have further refined lesion characterization by providing valuable insights into tissue perfusion, cellular density, and metabolite composition [20,21,22].

In contrast, MRE provides a non-invasive, quantitative biomechanical assessment by measuring shear wave propagation through tissues and generating stiffness maps that reflect mechanical properties [11,21]. This technique has demonstrated promising utility in head and neck imaging, particularly in differentiating benign from malignant salivary gland tumors, given that malignant lesions typically exhibit increased stiffness values due to desmoplastic reactions and altered extracellular matrix composition [10]. Moreover, MRE has the potential to complement conventional MR techniques within a multiparametric imaging framework, thereby enhancing diagnostic accuracy, and potentially improving clinical decision-making [14,17]. Importantly, in the context of radiotherapy, where the parotid gland is notably susceptible to radiation-induced damage, MRE may facilitate the early detection of structural changes and aid in mitigating complications such as xerostomia, which significantly impact patient quality of life [23,24,25,26,27].

This study aimed to assess the technical feasibility of applying a standard 2D gradient-recalled echo liver MRE protocol for the biomechanical evaluation of the parotid gland in healthy volunteers. The results demonstrated that the protocol reliably yielded stiffness measurements in all participants, underscoring the technical applicability of MRE even in small, anatomically complex structures such as the parotid gland.

To situate these findings within the broader scientific context, a comprehensive literature review was performed across multiple databases including PubMed/MEDLINE, Scopus, Web of Science, Google Scholar, Embase, and ResearchGate. Eligible studies were those investigating MRE applications in the head and neck region or focusing on elastography-specific driver configurations suitable for small anatomical structures. Although the number of published studies remains limited, the existing literature consistently supports the technical feasibility of parotid gland MRE and highlights the pressing need for methodological standardization.

The pioneering in vivo application was reported by Haberman et al. [15], who conducted MRE on 20 healthy volunteers using a 3T MRI scanner and a mechanical driver positioned directly on the parotid gland. Their reported mean shear modulus closely aligns with the findings of our study, providing strong external validation. Yeung et al. [8] further substantiated these observations by adapting a liver-specific driver for parotid imaging and confirming consistent, repeatable bilateral stiffness measurements in a small volunteer cohort.

Elsholtz et al. [5] employed elastography utilizing two occipital pressure drivers and demonstrated that parotid gland stiffness can be reliably measured without the necessity of direct driver placement on the gland itself. However, this study has been critically noted by reviewers for its limited clinical applicability, primarily due to a lack of pathological correlation and the absence of clear evidence supporting the utility of standardized parotid parenchymal stiffness values in clinical diagnostics. In contrast, Atamaniuk et al. [11] introduced a dedicated parotid-specific driver adapted from liver MRE hardware, achieving measurable stiffness values on a 1.5T scanner in a smaller sample size. Our investigation differs by employing clinically accessible, standardized hardware on a higher field strength scanner (3T) and including a larger, well-defined cohort of healthy volunteers, thereby offering practical insights into the real-world feasibility and reproducibility of parotid MRE.

The potential clinical value of parotid MRE is further supported by studies such as that by Bhatia et al. [28], who observed significantly elevated stiffness in salivary gland tumors relative to contralateral healthy tissue within the same patient. Despite their limited sample size, these findings suggest that MRE may more sensitively capture intra-patient pathological heterogeneity than conventional imaging modalities. Similarly, Bahn et al. [9] applied MRE to the thyroid gland with a dedicated electromagnetic driver, confirming the technique’s feasibility in small-volume tissues, though with limited success in reliably differentiating benign from malignant nodules.

Furthermore, recent work by Tanabe et al. demonstrated the quantitative assessment of parotid gland stiffness using shear wave elastography, highlighting variations related to age, gender, and internal tissue architecture [16]. Their findings highlight the potential of elastographic assessment for preoperative planning and objective evaluation of parotid pathology in the context of OMFS.

Several technical considerations emerged from our results. The ICC for mean stiffness was 0.638 (moderate agreement), while maximum stiffness reached 0.918 (excellent reproducibility), highlighting MRE’s reliability in assessing parotid gland mechanics. The lower ICC for mean stiffness likely reflects the subjectivity of ROI delineation based on anatomical MRI, emphasizing the need for improved, possibly automated, segmentation methods.

Our study has several inherent limitations that warrant careful consideration. Firstly, the assessment was limited to a single parotid gland per participant, which restricts the capacity for direct intra-individual and bilateral comparisons. Although our sample size is larger than in some earlier studies, it remains relatively modest and exhibits an imbalance in sex distribution, potentially impacting the generalizability of the findings. Furthermore, the application of a liver-specific 2D MRE protocol combined with a standard pneumatic driver, rather than a parotid-targeted mechanical driver, may have influenced shear wave propagation and penetration due to the parotid gland’s small size and intricate anatomical features. In addition, the manual and subjective delineation of ROIs based on anatomical MRI sequences introduces potential variability, which may partially explain the moderate interobserver agreement observed for mean stiffness measurements. Finally, the absence of pathological validation and longitudinal reproducibility assessments limits clinical interpretation.

Despite these challenges, our findings contribute meaningfully to the growing evidence base supporting MRE as a feasible and potentially valuable tool for salivary gland assessment. Integration of MRE with advanced multiparametric MRI approaches may facilitate more comprehensive lesion characterization, particularly in distinguishing benign from malignant pathologies [17,18,19,20,21]. Moreover, the role of MRE in detecting and quantifying radiation-induced changes in salivary gland tissue is gaining recognition. Given the parotid gland’s sensitivity to radiotherapy and its critical function in saliva production, early detection of radiation-induced fibrosis and functional impairment via MRE could significantly inform personalized treatment planning and improve patient outcomes by minimizing adverse effects such as xerostomia [22,23,24]. It may also provide valuable insight into other relevant clinical problems, including post-radiotherapy fibrosis and inflammatory conditions such as IgG4-related disease, autoimmune inflammation (e.g., Sjögren’s syndrome), and other parotid gland inflammatory disorders [26,27].

Future research should aim to address these limitations by incorporating larger, more diverse cohorts, employing bilateral gland evaluation, developing parotid-specific hardware, and validating measurement reproducibility over time to fully establish the clinical utility and diagnostic accuracy of parotid gland MRE. Additionally, future research efforts should focus on validating parotid MRE in larger, more heterogeneous populations encompassing neoplastic, inflammatory, and post-radiation conditions. Priorities include standardization of acquisition parameters, optimization of driver placement strategies, bilateral gland imaging, and assessment of longitudinal reproducibility. Additionally, the development of compact, ergonomically designed, parotid-specific drivers—such as the passive driver recently introduced at ISMRM 2023 [29]—will likely be instrumental in facilitating broader clinical translation of this technique.

Additionally, from a surgical perspective, MRE could provide valuable biomechanical data to support OMF. Quantitative stiffness maps may enhance preoperative planning, especially for tumor resection near critical neurovascular structures, and aid in monitoring post-treatment tissue remodeling. Recent advances in image guided OMFS emphasize the integration of quantitative imaging biomarkers for surgical navigation, and MRE may complement these approaches by providing objective stiffness information [16,30].

## 5. Conclusions

Magnetic Resonance Elastography is a non-invasive, reliable, and reproducible imaging technique that enables quantitative evaluation of the mechanical properties of soft tissues. Our study demonstrates that parotid gland stiffness can be accurately quantified using a standard 3 Tesla MRI system, thereby highlighting the potential of MRE as a quantitative “palpation” modality in head and neck imaging.

Future research should focus on comprehensive evaluation of parotid biomechanical changes across various pathological states, including benign and malignant lesions as well as inflammatory conditions. Additionally, clarifying MRE’s role in functional assessment and early detection of radiation-induced changes in patients undergoing radiotherapy is critical. Such investigations will contribute to personalized treatment planning and mitigation of side effects in head and neck oncology.

Moreover, integrating MRE with advanced MRI techniques to enhance diagnostic accuracy, alongside efforts to standardize protocols and validate reproducibility across centers, will be essential for widespread clinical adoption. Ultimately, MRE promises to become a vital component of multimodal imaging strategies aimed at improving individualized patient management and clinical outcomes in head and neck radiology.

## Figures and Tables

**Table 1:** MRI Parameters of the Standard 2D GRE MRE Protocol Used for Parotid Gland Evaluation.

Parameter	Value
**Magnetic field strength**	3T
**Sequence**	GRE MRE
**Orientation**	Axial
**Number of slices**	4
**Slice thickness**	10 mm
**Field of view (FOV)**	420 mm
**Phase FOV**	100% (420 mm)
**Base resolution/Acquisition matrix**	256 × 64
**Phase resolution**	25%
**Phase encoding direction**	A–P
**Acceleration factor (ASSET, SENSE, GRAPPA)**	2
**Echo time (TE)**	Shortest (approx. 20 ms)
**Repetition time (TR)**	50 ms
**Flip angle (FA)**	20°
**Number of breath-holds**	4 (end-expiration, one per slice)

**Table 2:** Mean and standard deviation of maximum and mean stiffness values (kPa) of the left and right parotid glands assessed independently by two radiologists.

	R1_Max ± SD kPa	R1_Mean ± SD kPa	R2_Max ± SD kPa	R2_Mean ± SD kPa
**Left**	1.743 ± 0.721	1.356 ± 0.190	1.950 ± 0.469	1.272 ± 0.224
**Right**	1.401 ± 0.165	1.118 ± 0.167	1.363 ± 0.174	1.196 ± 0.146

**Table 3:** Stiffness values (kPa) of parotid glands in male participants. Standard deviation is not applicable (N/A) for the right parotid gland due to a single measurement.

Localization	R1_Max ± SD kPa	R1_Mean ± SD kPa	R2_Max ± SD kPa	R2_Mean ± SD kPa
**Left**	2.354 ± 1.040	1.222 ± 0.205	2.350 ± 1.012	1.207 ± 0.034
**Right**	1.390 ± N/A	1.153 ± N/A	1.342 ± N/A	1.278 ± N/A

**Table 4:** Stiffness values (kPa) of parotid glands in female participants as assessed independently by two radiologists.

Localization	R1_Max ± SD kPa	R1_Mean ± SD kPa	R2_Max ± SD kPa	R2_Mean ± SD kPa
**Left**	1.689 ± 0.385	1.359 ± 0.174	1.832 ± 0.337	1.343 ± 0.133
**Right**	1.326 ± 0.159	1.099 ± 0.173	1.359 ± 0.189	1.133 ± 0.158

**Table 5:** Descriptive statistics (kPa) of mean and maximum stiffness values of the parotid glands in 21 healthy participants, including inter-observer agreement analysis.

Statistic	R1-Max	R1-Mean	R2-Max	R2-Mean
**Count**	0.021 kPa	0.021 kPa	0.021 kPa	0.021 kPa
**Mean**	1.595 kPa	1.210 kPa	1.563 kPa	1.147 kPa
**Std**	0.532 kPa	0.241 kPa	0.529 kPa	0.234 kPa
**Min**	0.959 kPa	0.571 kPa	1.051 kPa	0.701 kPa
**Max**	3.362 kPa	1.671 kPa	3.395 kPa	1.505 kPa
**Median**	1.436 kPa	1.241 kPa	1.438 kPa	1.149 kPa

**Table 6:** Published Parotid Gland Elastography Studies and Reported Stiffness Values.

Study (Year)	Participants/Patients	Organ/Region	Method/Equipment/Driver	Frequency	Measured Parameters	Mean Stiffness/Findings
Elsholtz et al., 2022 [5]	20 healthy volunteers	Parotid	3T MRI, multifrequency tomoelastography	25–50 Hz	SWS, φ	SWS mean: 0.97 m/s, φ mean: 0.59 rad
Yeung et al., 2013 [8]	10 healthy volunteers	Parotid	3T MRI, adaptive driver for parotid	25–50 Hz	G’ (shear modulus)	1.12 ± 0.48 kPa
Atamaniuk et al., 2024 [11]	10 healthy volunteers	Parotid	3T MRI, high-frequency passive driver	50–100 Hz	Shear modulus	1.25 ± 0.20 kPa
Habermann et al., 2005 [15]	5 healthy volunteers	Parotid	3T MRI, mechanical driver directly on gland	50 Hz	Shear modulus	1.29 ± 0.08 kPa
Tanabe et al., 2025 [16]	124 parotid glands	Parotid	2D-SWE	-	Shear wave elastography	11.32 ± 1.91 kPa (SWE)

## Data Availability

Data supporting the conclusions reported in this study are openly available. In cases where it is not possible to share research data publicly due to confidentiality, ethical or legal restrictions, the data may be provided upon reasonable requests by specifying the conditions of access.
